# Overexpression of *Nictaba*-Like Lectin Genes from *Glycine max* Confers Tolerance toward *Pseudomonas syringae* Infection, Aphid Infestation and Salt Stress in Transgenic *Arabidopsis* Plants

**DOI:** 10.3389/fpls.2016.01590

**Published:** 2016-10-25

**Authors:** Sofie Van Holle, Guy Smagghe, Els J. M. Van Damme

**Affiliations:** ^1^Laboratory of Biochemistry and Glycobiology, Department of Molecular Biotechnology, Ghent UniversityGhent, Belgium; ^2^Laboratory of Agrozoology, Department of Crop Protection, Ghent UniversityGhent, Belgium

**Keywords:** lectin, Nictaba, soybean, *Phytophthora sojae*, *Pseudomonas syringae*, *Myzus persicae*, *Aphis glycines*, salt stress

## Abstract

Plants have evolved a sophisticated immune system that allows them to recognize invading pathogens by specialized receptors. Carbohydrate-binding proteins or lectins are part of this immune system and especially the lectins that reside in the nucleocytoplasmic compartment are known to be implicated in biotic and abiotic stress responses. The class of Nictaba-like lectins (NLL) groups all proteins with homology to the tobacco (*Nicotiana tabacum*) lectin, known as a stress-inducible lectin. Here we focus on two Nictaba homologs from soybean (*Glycine max*), referred to as *Gm*NLL1 and *Gm*NLL2. Confocal laser scanning microscopy of fusion constructs with the green fluorescent protein either transiently expressed in *Nicotiana benthamiana* leaves or stably transformed in tobacco BY-2 suspension cells revealed a nucleocytoplasmic localization for the *Gm*NLLs under study. RT-qPCR analysis of the transcript levels for the Nictaba-like lectins in soybean demonstrated that the genes are expressed in several tissues throughout the development of the plant. Furthermore, it was shown that salt treatment, *Phytophthora sojae* infection and *Aphis glycines* infestation trigger the expression of particular *NLL* genes. Stress experiments with *Arabidopsis* lines overexpressing the *NLLs* from soybean yielded an enhanced tolerance of the plant toward bacterial infection (*Pseudomonas syringae*), insect infestation (*Myzus persicae*) and salinity. Our data showed a better performance of the transgenic lines compared to wild type plants, indicating that the NLLs from soybean are implicated in the stress response. These data can help to further elucidate the physiological importance of the Nictaba-like lectins from soybean, which can ultimately lead to the design of crop plants with a better tolerance to changing environmental conditions.

## Introduction

To successfully survive in their natural habitat, plants are capable of experiencing stress when they are confronted with adverse environmental conditions including drought, insect infestation or pathogen infection. Because plants cannot flee from these unfavorable conditions, they have developed a sophisticated protection system which enables them to recognize disadvantageous situations, alter hormone crosstalk and successfully cope with these adverse growth conditions (Jones and Dangl, 2006). The plant's innate immune system can recognize invading pathogens by a range of specialized cell-surface and intracellular receptors. It was shown that lectins are part of the plant's immune system since they can act as immune receptors and/or defense proteins (Peumans and Van Damme, 1995; Lannoo and Van Damme, 2014).

The class of plant carbohydrate-binding proteins or lectins is widespread within the plant kingdom and these proteins exhibit specificities toward endogenous as well as exogenous glycan structures (Van Damme et al., 2008). During the last decade, compelling evidence has been offered demonstrating that next to the classical lectins that reside mostly in the vacuole, there is a group of inducible cytoplasmic/nuclear lectins. The latter group of lectins is not easily detectable in plants under normal environmental conditions, but their expression level is increased after application of certain stressors (Van Damme et al., 2004; Lannoo and Van Damme, 2010). At present, at least six carbohydrate recognition domains have been identified within the group of nucleocytoplasmic lectins (Lannoo and Van Damme, 2010). Several of these nucleocytoplasmic lectins have been studied in detail and play roles in plant stress signaling (Al Atalah et al., 2014; Van Hove et al., 2015). One of these domains was first discovered in the *Nicotiana tabacum* (tobacco) agglutinin, abbreviated as Nictaba (Chen et al., 2002). In recent years, Nictaba was also shown to be implicated in the plant stress response (Chen et al., 2002; Lannoo et al., 2007; Vandenborre et al., 2009a, 2010; Delporte et al., 2011). This GlcNAc-binding lectin is believed to trigger gene expression in response to stress by interaction with the core histones H2A, H2B and H4 through their O-GlcNAc modification (Schouppe et al., 2011; Delporte et al., 2014).

An extensive survey of genome databases revealed that Nictaba-like lectins (NLLs) are widespread in plants (Delporte et al., 2015). Thus, far, functional characterization has been focused on the tobacco lectin and one F-box Nictaba homolog from *Arabidopsis* (Stefanowicz et al., 2012; Delporte et al., 2015). Lectin expression in tobacco is enhanced after caterpillar attack, suggesting a role for Nictaba in plant defense. Furthermore, experiments using transgenic tobacco plants overexpressing the lectin gene or plants with reduced expression indicated that Nictaba exerts insecticidal activity toward Lepidopteran pest insects (Vandenborre et al., 2010). The *Arabidopsis* F-box-Nictaba homolog is upregulated after treatment with salicylic acid and upon *Pseudomonas syringae* infection and overexpression of the gene in *Arabidopsis* plants confers increased tolerance to the pathogen (Stefanowicz et al., 2016). In order to refine our understanding of this specific group of nucleocytoplasmic lectins, we focus here on some Nictaba-like lectins from soybean. Soybean presents an exciting opportunity to investigate the stress inducibility of these proteins in an important crop species. Several *GmNictaba*-related genes have recently been identified in the soybean genome. Of the 31 identified *GmNLL* genes, 25 encode chimerolectins, consisting of one Nictaba lectin domain combined with an N-terminal F-box protein domain. The remaining six genes encode Nictaba orthologs containing one or two Nictaba domains as building blocks (Van Holle and Van Damme, 2015).

In this study, two *GmNLL* genes, referred to as *GmNLL1* and *GmNLL2*, located on different chromosomes have been selected for analysis. Their localization in the cell was investigated, together with their temporal and spatial expression in wild type soybean plants subjected to a variety of abiotic and biotic stresses. In addition, *Arabidopsis* overexpression lines were generated and analyzed for tolerance toward pathogen infection and aphid infestation. These data allowed us to investigate if overexpression of the *GmNictaba*-related genes leads to an enhanced tolerance of the plant toward stress.

## Materials and methods

### Plant materials and growth conditions

Wild type seeds of *Arabidopsis thaliana* ecotype Colombia were purchased from Lehle Seeds (Texas, USA). For *in vitro* cultures, seeds were surface sterilized by submergence in 70% ethanol for 2 min, followed by 10 min in 5% NaOCl. Finally, the seeds were rinsed four to five times with sterilized water. *In vitro* cultures were maintained in a plant growth room at 21°C and a 16/8 h light/dark photoperiod. *Arabidopsis* plants were sown into Jiffy-7® (artificial soil) and grown in a Conviron (Berlin, Germany) plant growth cabinet under 12/12 h light/dark conditions at 21°C after stratification at 4°C for 3 days. Seeds for the insect assays were sown in round plastic pots (diameter: 11 cm) containing soil. After stratification pots were moved to a plant growth incubator (MLR-352 incubator, Sanyo/Panasonic, Osaka, Japan, 21°C, 12 h photoperiod, 75% relative humidity).

*Glycine max* cv Williams seeds were obtained from the USDA Soybean Germplasm Collection in Urbana (IL, USA). *Glycine max* cv Opaline seeds were obtained from the Institute for Agricultural and Fisheries Research (Merelbeke, Belgium). Seeds were grown in pots containing a mixture (50/50) of commercial soil and expanded clay granules (Agrex) in a growth chamber at 26°C with a 16/8 h light/dark photoperiod.

*Nicotiana benthamiana* seeds were kindly supplied by dr. Verne A. Sisson (Oxford Tobacco Research Station, Oxford, NC, USA). *N. benthamiana* plants were sown in pots containing commercial soil and grown in a growth chamber at 26°C with a 16/8 h light/dark photoperiod. The *N. tabacum* cv Bright Yellow-2 (BY-2) cell suspension culture was obtained from the department of Plant Systems Biology (Flanders Institute for Biotechnology, Zwijnaarde, Belgium) and maintained as described by Delporte et al. (2014).

### Pathogens

*Phytophthora sojae* was obtained from the CBS-KNAW Fungal Biodiversity Centre (Utrecht, The Netherlands) and was routinely cultured on 10% clarified and buffered V8-juice agar plates at 21°C in the dark. *Phytophthora brassicae* was grown under the same conditions and was kindly provided by Prof. Monica Höfte (Dept. of Crop Protection, Ghent University). *Pseudomonas syringae* pv. *tomato* strain DC3000 was also provided by Prof. Monica Höfte (Dept. of Crop Protection, Ghent University) and grown on King's B agar medium supplemented with 50 μg/ml rifampicin.

### Cloning of the *Nictaba*-like sequences from soybean

Trifoliate leaves from 18-day-old soybean (*Glycine max* cv Williams) plants were collected for RNA extraction. Total RNA was extracted using TRI Reagent® according to the manufacturer's instructions (Sigma-Aldrich). Residual genomic DNA was removed by a DNase I treatment (Life Technologies, Carlsbad, CA, USA) and RNA was quantified with a NanoDrop 2000 spectrophotometer (Thermo Fisher Scientific, Waltham, MA, USA). Reverse transcriptase reactions were performed with 1 μg of total RNA using moloney murine leukemia virus reverse transcriptase (M-MLV RT) and oligo(dT)25 primers (Life Technologies). The full length cDNA sequences corresponding to *NLL1* (*Glyma.06G221100*) and *NLL2* (*Glyma.20G020900*) were obtained by RT-PCR reactions with gene specific primers (Supplementary Table 1). Finally, the PCR products were ligated in the pJET2.1 vector with the CloneJET PCR Cloning kit according to the manufacturer's instructions (Life Technologies) and constructs were sequenced (LGC Genomics, Berlin, Germany) to confirm the cDNA sequence of the *GmNLL* genes.

### Construction of expression vectors

Vectors for expression of each of the *GmNLL* sequences either N- or C-terminally linked to EGFP (enhanced green fluorescent protein) under control of the CaMV 35S promoter were constructed using Life Technologies' Gateway® Cloning Technology. First, the cDNA clones were used as template in two consecutive PCRs and amplified with primers to attach *att*B sites to the PCR product. In the first PCR, the coding sequence of the *GmNLLs* was amplified using Platinum® *Pfx* DNA Polymerase (Life Technologies) and primers with stop codon (evd1022/evd1032 (*NLL1*) and evd1024/evd1033 (*NLL2*)) or without stop codon (evd1022/evd1023 (*NLL1*) and evd1024/evd1025 (*NLL2*)) (Supplementary Table 2) using the following cycling parameters: 2 min at 94°C, 25 cycles (15 s at 94°C, 30 s at 48°C, 1.5 min at 68°C), 5 min at 68°C. In the second PCR primers evd2/evd4 were used to complete the *att*B sites using following cycling parameters: 2 min at 94°C, 5 cycles (15 s at 94°C, 30 s at 48°C, 1.5 min at 68°C), 25 cycles (15 s at 94°C, 30 s at 55°C, 1.5 min at 68°C), 5 min at 68°C. The PCR products were used as substrates in a BP recombination reaction with the pDONR221 donor vector. Subsequently, the entry clones were recombined with destination vectors pK7WGF2,0 and pK7FWG2,0 to create the desired expression clones to create N- or C-terminal EGFP fusions to the *NLL* gene sequences, respectively (Karimi et al., 2002). Using a similar approach, coding sequences of *GmNLL1* and *GmNLL2* were introduced into the binary vector pK7WG2,0 (Karimi et al., 2002) to generate expression vectors for transformation of *Arabidopsis* plants.

The binary vectors carrying the different constructs were introduced into *Agrobacterium tumefaciens* C58C1 Rif (pGV4000) using the freeze/thaw transformation method. Briefly, 1 μg of the expression clones was added to competent *A. tumefaciens* cells followed by an incubation of 30 min on ice. Next, the cells were frozen in liquid nitrogen, thawed at 37°C for 5 min, and after addition of 1 ml of preheated LB medium, the cells were incubated for 2 h at 26°C. Transformed cells were selected on LB agar plates containing 50 μg/ml spectinomycin and screened by colony PCR.

### Transformation of *N. benthamiana* plants and *N. tabacum* cv BY-2 cells

Transient expression of the EGFP fusion proteins was conducted as described by Sparkes et al. (2006). The abaxial epidermis of young leaves of 4- to 6-week-old *N. benthamiana* plants was infiltrated with the *Agrobacterium* suspension harboring the different constructs. Two days post-infiltration, the infiltrated leaf areas were cut and analyzed microscopically. The tobacco BY-2 cell suspension culture was stably transformed with the EGFP fusion constructs under the control of the 35S promoter as described by Delporte et al. (2014).

### Generation of *Arabidopsis* transgenic lines

*Arabidopsis* 35S::*GmNLL1* and 35S::*GmNLL2* overexpression lines were generated using the floral dip method (Clough and Bent, 1998). Transformed seeds were selected using the adapted protocol proposed by Harrison et al. (2006). Integration of the T-DNA was detected by RT-PCR on cDNA with gene specific primers (Supplementary Table 3) using the following PCR program: 5 min at 95°C, 40 cycles of 45 s at 95°C, 45 s at 60°C, and 30 s at 72°C and a final 5 min at 72°C. Relative expression levels of the *GmNLL* genes were analyzed in 4-week-old plants by RT-qPCR. At least three independent homozygous single insertion lines of 35S::*GmNLL1* and 35S::*GmNLL2* were selected and used in all experiments, together with the corresponding wild type plant.

### Hormone treatment and abiotic stress application of wild type soybean plants

For hormone and salt stress treatments, 14-day-old soybean (*Glycine max* cv Williams) plants (V1 growth stage) were carefully removed from the soil and transferred to liquid Murashige and Skoog (MS) medium containing different hormones (100 μM abscisic acid (ABA), 50 μM methyl jasmonate (MeJA) or 300 mM salicylic acid (SA)) or 150 mM NaCl. For control treatments, equal volumes of the dissolvent (ethanol or water) of the hormone or salt solution were added to the medium. Treated root and shoot tissues were sampled at the following time points: 3, 6, 10, 24, and/or 32 h. Likewise, the corresponding mock controls were sampled at each time point. Plant material of four individual plants was pooled for each sample and immediately frozen in liquid nitrogen and stored at −80°C until use. In total, three biological replicates were performed.

### Infection assays of wild type soybean plants

Infection assays with *P. sojae* on wild type soybean plants were performed by inoculating fresh mycelial plugs (0.5 cm diameter) on the abaxial side of detached leaves of 10-day-old soybean plants (*Glycine max* cv Opaline). Mock infections included inoculation with blank V8-agar plugs. The petioles of the detached leaves were wrapped in cotton wool and the inoculated plants were placed in a tray containing three layers of wetted absorbent paper and closed with plastic wrap foil to maintain a relative humidity of 100%. Treatments and controls were incubated in a growth room at 26°C with a 16/8 h light/dark photoperiod. Samples were collected 1, 3, and 5 days post-infection and leaves of three individual plants per treatment were pooled at each time point. Three individual biological replicates were performed.

### Insect maintenance and non-choice experiment with wild type soybean

*Aphis glycines* (soybean aphid) was kindly provided by dr. Annie-Eve Gagnon (CÉROM, Quebec, Canada) and reared on soybean plants under standard conditions in a growth incubator (MLR-352 incubator, Sanyo/Panasonic, Osaka, Japan) at 25°C, 60% relative humidity and a 16 h photoperiod. In a non-choice experiment, the first trifoliate leaves of 14-day-old soybean plants were placed in a cage (Novolab) with 60 apterous adult aphids. Control samples included the cage without aphids. Three leaves from individual plants of treated and control plants were harvested and pooled after the designated time points (3, 5, and 7 days), and snap frozen in liquid nitrogen. Three individual biological replicates were performed.

### Real-time quantitative RT-PCR

For gene expression analysis, all collected leaf and root samples were ground in liquid nitrogen and stored at −80°C until further analysis. RNA extraction was performed using TriReagent® (Sigma-Aldrich). Next, a DNAse I treatment (Life Technologies) was performed and the RNA concentration and quality was assessed spectrophotometrically. First-strand cDNA was synthesized from 1 μg of total RNA with oligo(dT)25 primers and 200 U of M-MLV reverse transcriptase (Life Technologies). Subsequently, the cDNA was diluted 2.5 times and cDNA quality was checked by RT-PCR with SKP1/Ask-interacting protein 16 primers (SKIP16). Quantitative RT–PCR was performed with the 96-well CFX Connect™ Real-Time PCR Detection System (Bio-Rad) using the SensiMix™ SYBR® No-ROX One-Step kit (Bioline Reagents Limited, London, UK). Reactions were conducted in a total volume of 20 μl containing 1 × SensiMix™ SYBR® No-ROX One-Step mix, 500 nM gene specific forward and reverse primer and 2 μl cDNA template. RT-qPCR was performed under following conditions: 10 min at 95°C, 45 cycles of 15 s at 95°C, 25 s at 60°C, and 20 s at 72°C and a melting curve was generated after every RT-qPCR run. Independent biological replicates and technical replicates were analyzed together using the sample maximization approach (Hellemans et al., 2007). An overview of all primers used in the qPCR analyses can be found in Supplementary Table 3 and the reference genes for each experiment are listed in Supplementary Table 4. Based on the available literature, different reference genes were selected because they were demonstrated to be the most stable under certain conditions. Melting curve analysis was performed after each run (Bio-Rad CFX Manager 3.1 software). Reference gene stability and quality control of the samples were validated in the qBASEPLUS software (Hellemans et al., 2007) and the results were statistically evaluated with the REST-384 software using the pair wise fixed reallocation randomization test (with 2000 randomizations; Pfaffl et al., 2002). Gene specific primers were designed using Primer3 (http://biotools.umassmed.edu/bioapps/primer3_www.cgi) and the specificity (BLAST search) and presence of SNPs were analyzed *in silico*, next to the secondary structure evaluation of the amplicon (Derveaux et al., 2010). Gene specific primers were evaluated by verification of the amplicon and determination of the amplification efficiency.

### Germination assays

For the seed germination assay, seeds of wild type plants and four independent homozygous transgenic lines for each construct (35S::*GmNLL1* and 35S::*GmNLL2*) were grown on ½ MS medium (Duchefa Biochemie, Haarlem, The Netherlands) containing 50 or 150 mM NaCl (50 seeds/line/treatment). After the stratification for 3 days at 4°C in the dark, the plates were placed in a plant growth room at 21°C and a 16/8 h light/dark cycle. Germination was assigned as the emergence of the radicle through the seed coat. Germination on ½ MS medium without additional NaCl was performed as a control. Two biological replicates were performed with 50 plants per line for each treatment.

To determine post-germination growth, plants were sown on ½ MS medium and after the stratification (3 days at 4°C in the dark), the plants were grown at 21°C in a plant growth room with a 16/8 h light/dark cycle. Seven-day-old plantlets were transferred to ½ MS medium with 50 or 150 mM NaCl and after 1 week, the percentage of discolored leaves was determined. Chlorophyll was extracted by adding 10 ml N,N-dimethylformamide to the leaf material and after a 2 h incubation, the absorbance of the supernatant was measured at 645 and 663 nm. Chlorophyll *a* and *b* were determined as described by Porra (2002): [Chl *a*] = 12 A_663_ − 3.11 A_645_, [Chl *b*] = 20.78 A_663_ − 4.88 A_645_, and [Chl *a* + *b*] = 17.67 A_663_ + 7.12 A_645_. Two biological replicates were performed with 50 plants per line for each treatment.

### Root growth analysis

The root growth assay was performed as follows: 30 seeds of wild type plants and the different overexpression lines were germinated on ½ MS medium supplemented with 0, 50, or 150 mM NaCl. Plates were kept in the dark for 3 days at 4°C to break seed dormancy and were then transferred to a plant growth room at 21°C and long day (16/8 h light/dark) growth conditions. Primary root length of 2-week-old plantlets was determined with Root Detection 0.1.2 (http://www.labutils.de/rd.html). The experiment was repeated twice.

### Non-choice aphid experiment with *Arabidopsis*

A permanent colony of the green peach aphid (*Myzus persicae*) was kept on sweet pepper plants under standard lab conditions (Shahidi-Noghabi et al., 2009). In a non-choice infection assay, five adult aphids were collected from rearing plants and placed on 4-week-old *Arabidopsis* leaves with a brush. After 4 days, all adult aphids were removed from the plants and the plants were returned to the plant growth incubator. On day 8, the plants were harvested and the number of nymphs and aphids residing on each plant was counted. This experiment was repeated twice with six individual plants of each line in each of the experiments.

### *Phytophthora* infection assay of *Arabidopsis*

Adult rosette leaves from 4-week-old *Arabidopsis* plants were drop inoculated with 20 μl *P. brassicae* zoospore solution (10^5^ spores/ml) or mock inoculated with water. The zoospore solution was initiated as described by Bouwmeester and Govers (2009). Upon inoculation, the plants were kept in the growth cabinet under 100 % relative humidity. Samples were taken at 1, 3, 5, and 10 dpi.

Plant inoculation with pathogen mycelia was performed by placing fresh mycelium agar plugs (0.5 cm diameter) onto ½ MS agar plates without sugar. Two-week-old *in vitro* grown *Arabidopsis* plants were placed next to the pathogen and susceptibility was evaluated 14 days post-inoculation. Mock inoculations were performed with clean V8-agar plugs.

### *Pseudomonas syringae* infection assay of *Arabidopsis*

*Pseudomonas* infection assays with transgenic *Arabidopsis* plants were performed as described previously with some modifications (Pieterse et al., 1996; Katagiri et al., 2002). Four-week-old *Arabidopsis* plants were spray-inoculated with the *Pseudomonas* suspension (1.6 × 10^7^ CFU/ml in 10 mM MgSO_4_ and 0.05% Silwet-L77) or mock inoculated with 10 mM MgSO_4_ and 0.05% Silwet-L77. During the first 72 h after inoculation, plants were kept in 100 % relative humidity in a Conviron plant growth cabinet (Berlin, Germany). Leaves of three individual plants were sampled at 1, 2, 3, 4, and 5 dpi. Two biological replicates were performed. To estimate the lesion area, leaves were scanned with a flatbed scanner at the highest resolution. Lesion areas of individual leaves were determined in the Image Analysis Software for Plant Disease Quantification Assess 2.0 (APS, St. Paul, USA) using a self-written macro.

*Arabidopsis* leaves inoculated with *P. syringae* collected at 3 and 4 dpi were used for genomic DNA extraction. DNA from approximately 100 mg of plant material was extracted using a CTAB buffer (2% CTAB, 0.1 M Tris/HCl pH 7.5; 1.4 M NaCl; 2 mM EDTA), followed by a chloroform:isoamyl alcohol (24:1) extraction. DNA was precipitated with 100% isopropanol and washed with 76% EtOH/0.2 M NaOCl and 76% EtOH/10 mM NH_4_OAc. The *oprF* primers were used to target the outer membrane porin protein F gene of *P. syringae* (Brouwer et al., 2003) and *Act2* and *PEX4* primers were used as endogenous controls for *Arabidopsis* (Supplementary Table 3). The ratio of *P. syringae* genomic DNA to *Arabidopsis* DNA was calculated using REST-384 software (Pfaffl et al., 2002). Two biological replicates with two technical replicates were analyzed.

### Confocal microscopy and image analysis

Images were acquired with a Nikon A1R confocal laser scanning microscope (Nikon Instruments) mounted on a Nikon Ti-E inverted epifluorescence body with an S Plan Fluor ELWD 40 × Ph2 ADM objective (NA 0.60). Different fluorescent images were acquired along the z-axis to create a picture of the complete cell. EGFP was excited with a 488 nm argon ion laser and a 515–530 nm emission filter was used. Image analysis was conducted in Fiji (Schindelin et al., 2012) and the JaCoP tool (Bolte and Cordelières, 2006) was used for colocalization analysis.

### Online tools

Prediction of protein subcellular localization and signal peptide were performed with the TargetP 1.1 and SignalP 4.1 server, respectively (Emanuelsson et al., 2000; Petersen et al., 2011). BLAST searches were conducted on the Phytozome website (https://phytozome.jgi.doe.gov/pz/) using default settings. Multiple sequence alignments and pairwise sequence alignments were performed with ClustalO 1.2.1 (http://www.ebi.ac.uk/Tools/msa/clustalo/) and EMBOSS Water (http://www.ebi.ac.uk/Tools/psa/emboss_water/), respectively. Normalized RNA-sequencing data was downloaded on the SoyBase website (http://soybase.org/soyseq/) (Severin et al., 2010).

### Statistical analysis

Statistical analysis was conducted using SPSS Statistics 22 (IBM) and the data were considered statistically significant for *p* < 0.05. The assumption of normality was tested with the Shapiro-Wilkinson test and the equality of variances of normally distributed data was assessed using the Levene's test. The Welch and Brown-Forsythe tests were performed when the homogeneity of variance of the data was invalid. ANOVA was used to determine statistically significant differences between groups with normally distributed data. For not-normally distributed samples, the Mann-Whitney *U*-test was performed, supplemented with the non-parametric Levene's equivalent to test homogeneity of variance. Tukey was used as *post-hoc* test with Bonferroni-Holm correction for multiple testing. This correction was also applied for Mann-Whitney tests between different groups. Data with a binomial distribution were subjected to Pearson's chi-square test. All results are shown as the mean ± SE (^*^p < 0.05, ^**^p < 0.01, ^***^p < 0.001).

## Results

### The nictaba-like lectins from soybean show high sequence similarity to nictaba

**Figure 1:**
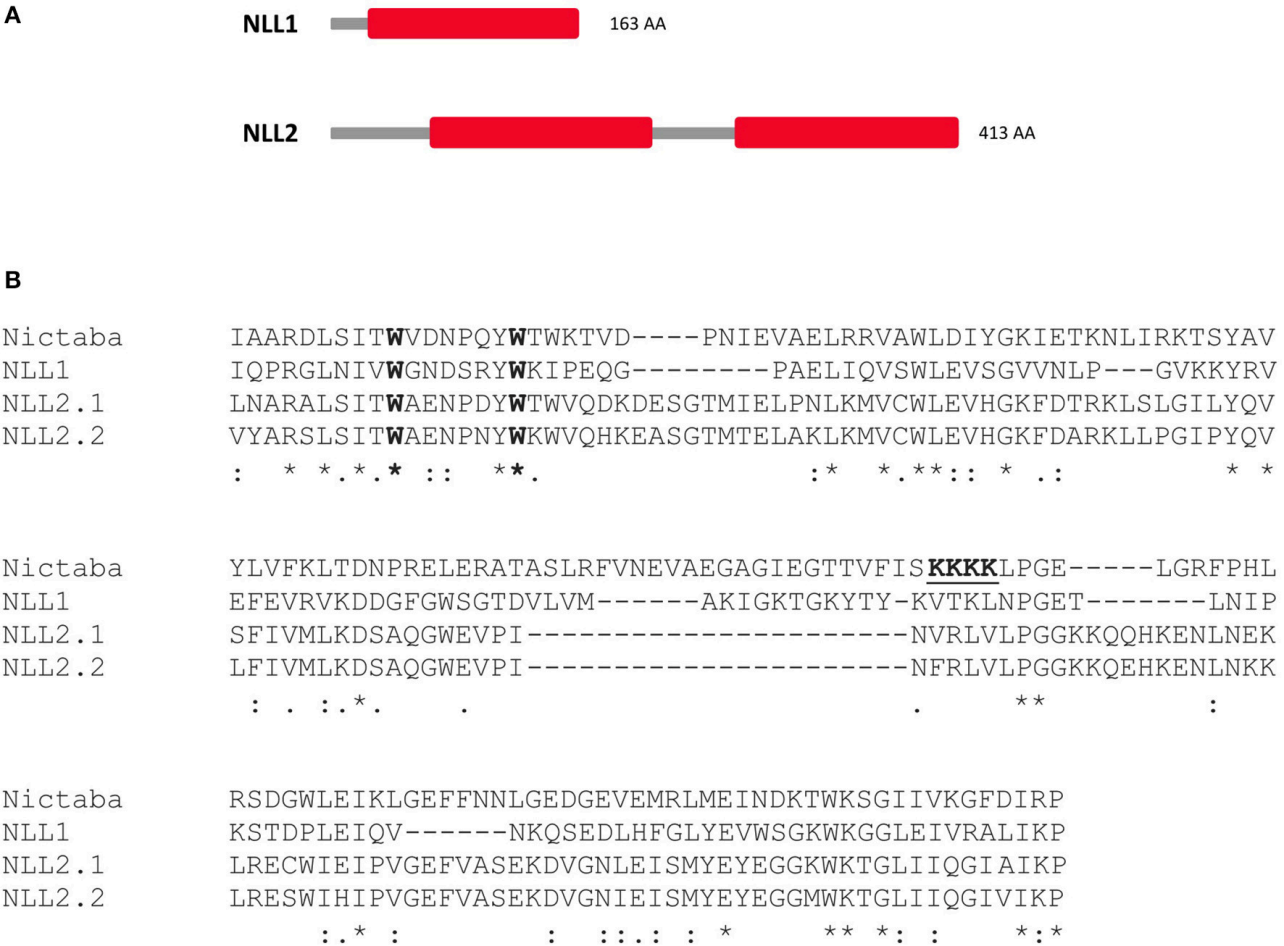
**(A)** Domain architecture of the Nictaba-like homologs from soybean under study: NLL1 (*Glyma.06G221100*) and NLL2 (*Glyma.20G020900*) **(B)** Sequence alignment of the trimmed Nictaba sequence and the Nictaba domains of NLL1 and NLL2 (NLL2.1: domain one, NLL2.2: domain two) from soybean using ClustalO. The conserved Trp-residues important for the carbohydrate-binding activity of Nictaba are marked in bold and the proposed nuclear localization signal of Nictaba is underlined. ‘*’indicates fully conserved amino acid residue; ‘:’designates conserved amino acid substitution (indicating conservation between groups of strongly similar properties); ‘.’designates semi-conserved amino acid substitution (indicating conservation between groups of weakly similar properties).

In a previous study 31 genes with homology to the *Nictaba* gene from tobacco have been identified in the soybean genome (Van Holle and Van Damme, 2015). Six of them are composed of one or more Nictaba domains, and two of these genes, designated as *GmNLL1* and *GmNLL2*, were selected for further study. Sequence comparison between the amino acid sequences from Nictaba (encoded by AF389848) and the two Nictaba-like proteins from soybean showed that these sequences are highly related. In contrast to the tobacco lectin sequence, which only consists of a Nictaba domain, the Nictaba domain from *Gm*NLL1 is preceded by an N-terminal domain of 24 amino acids. The *Gm*NLL2 sequence encodes an N-terminal domain of 66 amino acids followed by two Nictaba domains separated by a 51 amino acid linker (Figure 1A). BLASTp searches revealed that the N-terminal sequences of NLL1 and NLL2 show no sequence homology to any other plant protein.

Amino acid sequence alignment of Nictaba with the Nictaba domains of the *Gm*NLLs revealed 26 and 39% sequence identity, and 39 and 48 % sequence similarity for NLL1 and NLL2, respectively. Additionally, the two Trp residues which are imperative for the carbohydrate-binding activity of the tobacco lectin (Schouppe et al., 2010), are conserved in the soybean Nictaba homologs (Figure 1B). The putative nuclear localization signal sequence (^102^KKKK^105^) present in the Nictaba sequence was not conserved in the *Gm*NLL sequences (Figure 1B).

### The nictaba-like lectins from soybean localize to the nucleus and cytoplasm

**Figure 2:**
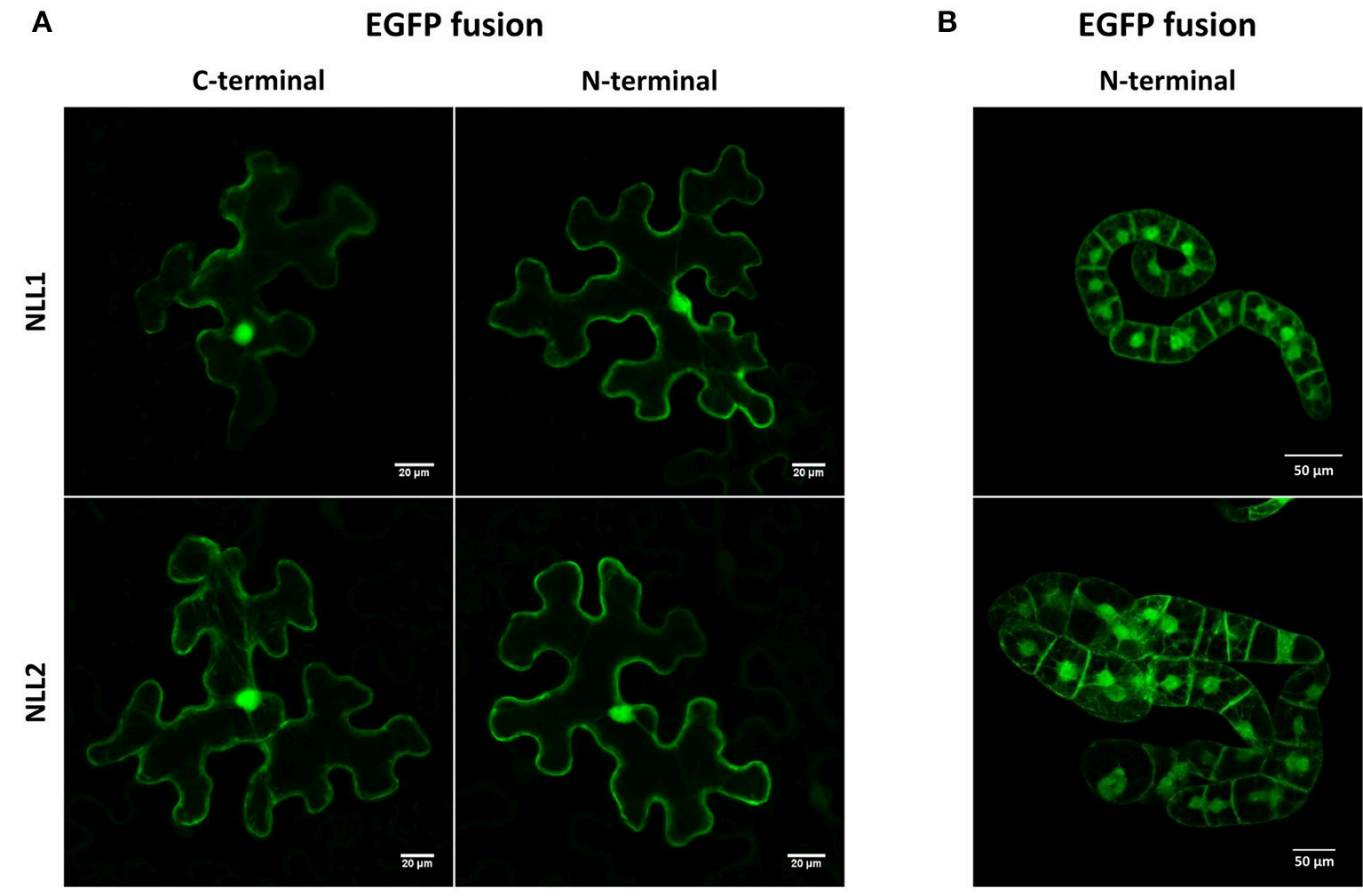
**Localization pattern of N- and C-terminal EGFP fusion constructs expressed in (A) transiently transformed *N. benthamiana* leaves and (B) in stably transformed BY-2 cells**.

Analysis of the *Gm*NLL sequences using the SignalP 4.1 server (Petersen et al., 2011) indicated the absence of a signal peptide, suggesting that these proteins are synthesized on free ribosomes and reside in the cytoplasm. Since the TargetP 1.1 software (Emanuelsson et al., 2000) did not allow a clear prediction of the subcellular localization for the GmNLLs, fusion constructs of the *GmNLL* coding sequences N- or C-terminally tagged with EGFP were used for transient expression in *N. benthamiana* leaves. Confocal microscopy of leaf tissue at day 2 post-infiltration revealed fluorescence in the nucleus and the cytoplasm of the epidermal cells, with similar images for the N- and C-terminal EGFP fusion constructs for NLL1 and NLL2 (Figure 2A). Similar localization patterns were obtained after stable transformation of tobacco BY-2 suspension cells confirming that GmNLL1 and GmNLL2 localize to the nucleus and the cytoplasm (Figure 2B).

### Expression of *NLL* genes during soybean development

**Figure 3:**
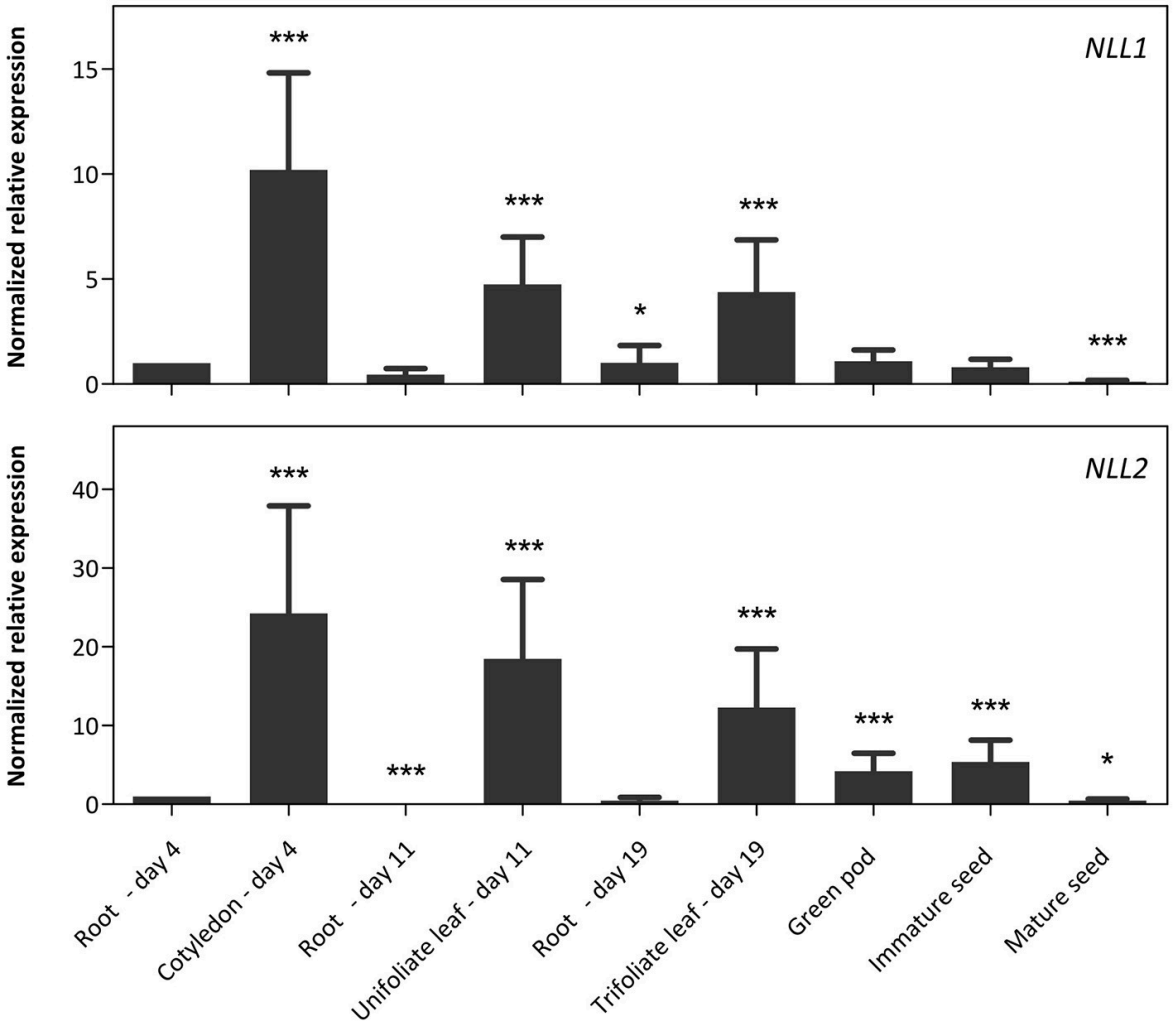
**Normalized relative expression profile of the three *GmNLL* genes during the development of the soybean plant**. The normalized transcript levels of all genes are represented relative to the expression of 4-day-old roots. The data represent three independent biological replicates, error bars indicate standard errors and statistically significant differences to the expression level of 4-day old roots are indicated with asterisks (^*^p < 0.05, ^***^p < 0.001).

To investigate the expression level of the *NLL* genes in different tissues from soybean, plants were grown under normal growth conditions and different tissue samples were taken from day four after sowing until maturity of the seed pods. Transcript levels for *GmNLLs* and some classical lectins of the legume lectin family were quantified using RT-qPCR and the expression was compared between different tissues (Figure 3).

The transcript levels for the *NLL1* gene are the highest in the cotyledons, unifoliate, and trifoliate leaves, but are significantly lower in belowground and reproductive tissues. The expression profile for the *NLL2* gene resembles that of *NLL1* with high expression in the leaves and significantly lower expression in roots. Yet, the *NLL2* transcript levels in green pods and immature seeds are higher compared to the transcript level of roots at day 4. Based on the raw Cq values of the different genes in the different samples, the expression level of the *NLL1* gene corresponds well to the expression level of the three reference genes while transcript levels for *NLL2* are less abundant than the *NLL1* gene and the reference genes (Supplementary Table 5).

**Figure 4:**
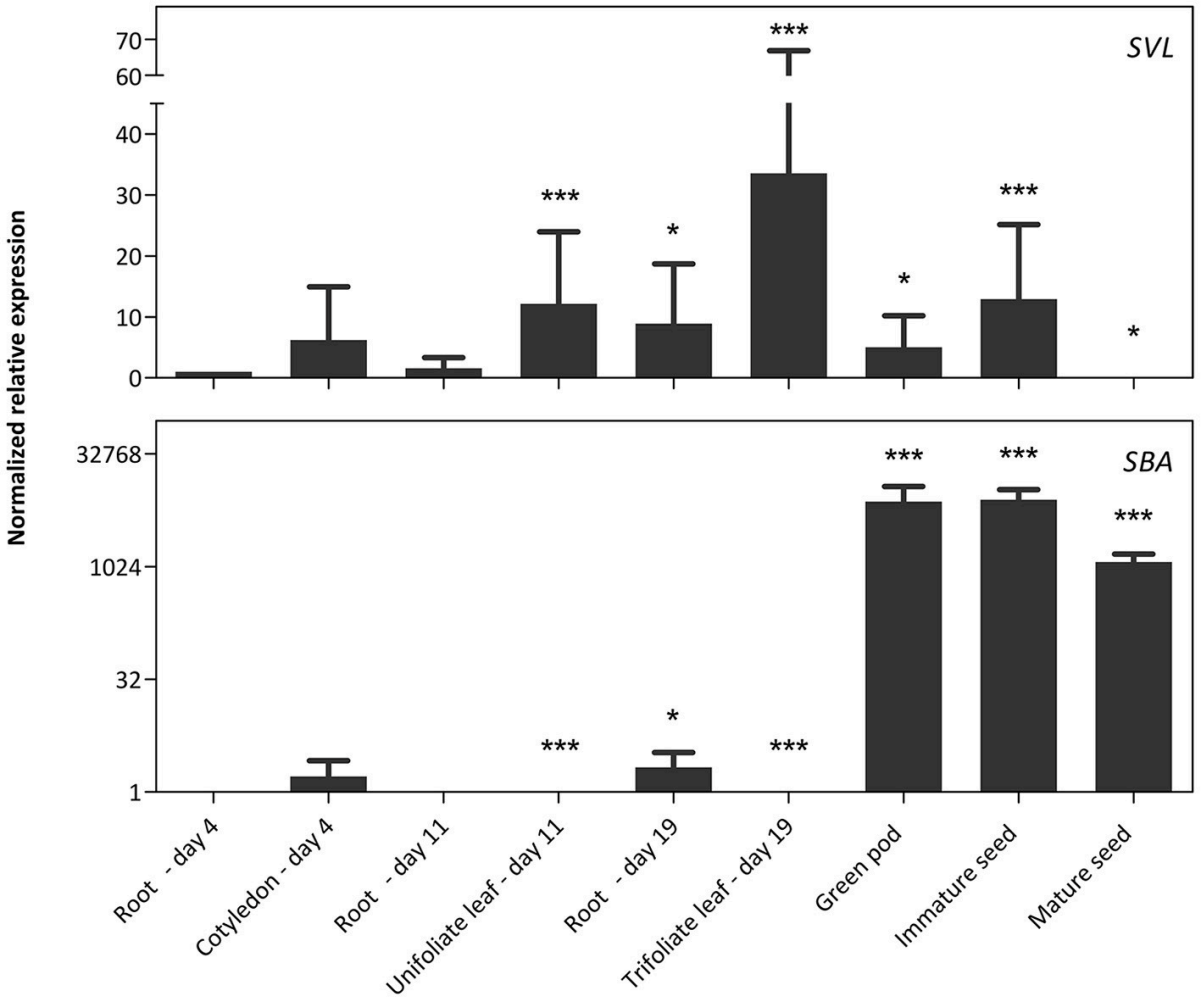
**Normalized relative expression profile of *SVL* and *SBA* during soybean development**. The normalized transcript levels of all genes are represented relative to the expression of 4-day-old roots. The data represent three independent biological replicates, error bars indicate standard errors and statistically significant differences to the expression level of 4-day-old roots are indicated with asterisks (^*^p < 0.05, ^***^p < 0.001).

The RT-qPCR analysis for the *NLL1* and *NLL2* genes was complemented with a comparative analysis to the *SVL* (soybean vegetative lectin) and *SBA* (soybean agglutinin) genes, two previously identified legume lectin genes from soybean (Figure 4). The transcript levels for *SVL* are the highest in leaves but lower transcript levels were also detected in green pods, immature seeds and roots of 19-day-old plants. In contrast very high transcript levels for the *SBA* gene were observed in pods and seeds. The expression is higher in green pods and immature seeds, compared to mature seeds. Considerably lower transcript levels of the *SBA* gene were detected in young cotyledons and in 19-day-old roots.

### *Nictaba*-like genes are stress inducible in soybean

**Figure 5:**
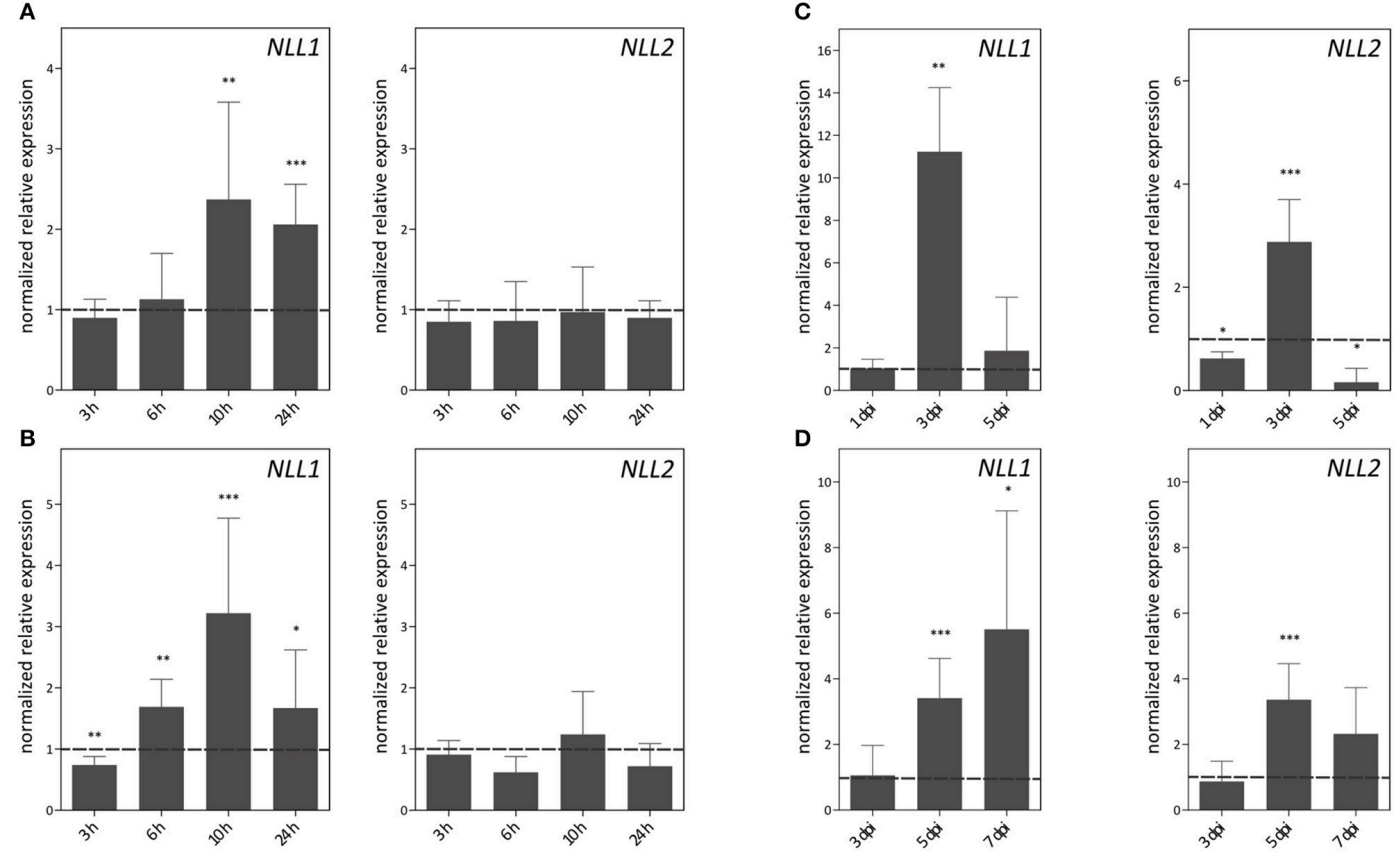
**Expression patterns of *GmNLL1* and *GmNLL2* under different stress conditions, determined by RT-qPCR**. Expression patterns under salt stress on leaf **(A)** and root **(B)** material; **(C)** transcript levels in leaf material upon *Phytophthora sojae* infection and **(D)** *Aphis glycines* infestation. The normalized expression levels, relative to the control treatment (set to 1) at the indicated time points are shown. The mean values of RT-qPCR from three independent biological replicates were normalized to three reference genes and error bars indicate standard errors. Asterisks indicate statistically significant differences compared to the control treatment (^*^p < 0.05, ^**^p < 0.01, ^***^p < 0.001).

The expression patterns of *GmNLL1* and *GmNLL2* were investigated in shoots and roots of 14-day-old plants subjected to different stress treatments. The RT-qPCR data reveal that salt treatment, *P. sojae* infection and *A. glycines* infestation trigger the expression of particular *NLL* genes (Figure 5). Interestingly, the expression of the two *GmNLLs* displayed dissimilar patterns under each of the different stress treatments. Salt stress conditions triggered the transcription of the *NLL1* gene in leaves and roots (Figures 5A,B). Transcript levels in both leaves and roots reached a peak 10 h after the start of the treatment. Gene expression levels of *NLL2* in leaves and roots were not altered by salt treatment. Infection with *P. sojae* (Figure 5C) triggered both *GmNLL1* and *GmNLL2* gene expression. The upregulation of *GmNLL1* and *GmNLL2* was the highest at 3 days post-infection, being approximately 11 and 3-fold higher than the non-treated plants for *NLL1* and *NLL2*, respectively. After aphid infestation, the expression of *NLL1* and *NLL2* showed an upregulation at 5 and/or 7 days post-infection. Compared to the expression level of *NLL1, NLL2* was triggered to a lower extent (Figure 5D). Application of the hormones ABA and MeJA did not greatly influence the transcript levels for *GmNLL1 or GmNLL2*. During SA treatment, the relative expression levels of *GmNLL1* and *GmNLL2* in root tissues were decreased significantly, suggesting that these gene products are not required in the plant's response upon SA treatment. The transcript levels of *GmNLL1* and *GmNLL2* in leaf tissues were not impacted by treatment with SA (Supplementary Figure 1). Our data show a differential expression pattern for the two *NLL* genes in both shoot and/or root tissues upon application of biotic or abiotic stresses, suggesting that these genes might play distinct roles in the plant.

### Overexpression of *GmNLL1* and *GmNLL2* in *Arabidopsis* confers increased tolerance to salt stress

**Figure 6:**
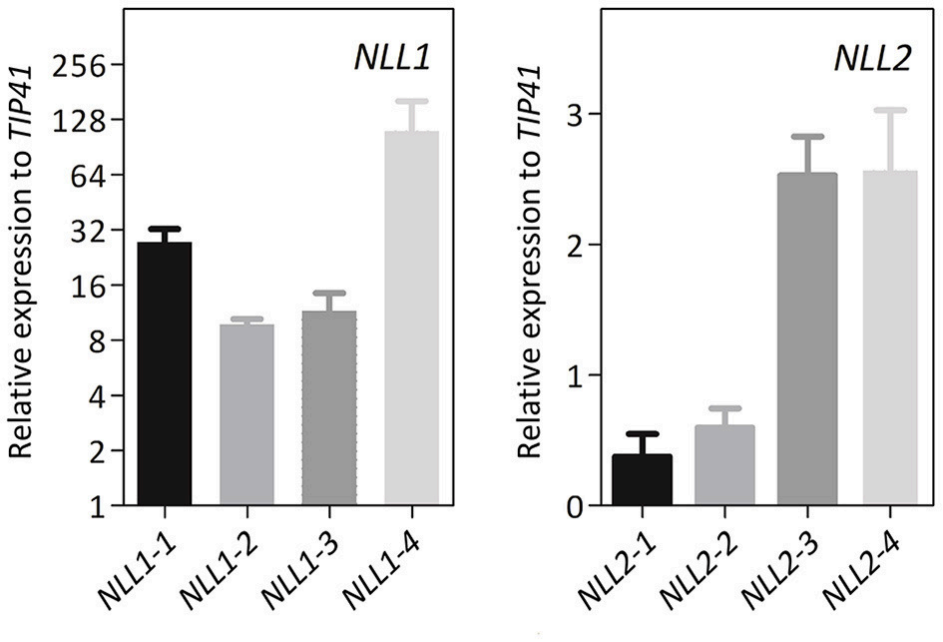
**Gene expression analysis of 4-week-old transgenic *Arabidopsis* plants overexpressing *GmNLL1* or *GmNLL2***. Normalized relative expression to reference gene *TIP41* of two biological replicates is represented (error bars represent standard errors).

To further investigate the biological function of the *Gm*NLLs, transgenic *Arabidopsis* lines that overexpress *GmNLL1* or *GmNLL2* driven by the CaMV 35S promoter were generated. Several independent homozygous lines carrying a single copy of the T-DNA insertion were screened and transcript levels for *GmNLL1* and *GmNLL2* were determined by RT-qPCR in 4-week-old plants. The transcript levels relative to the expression of *TIP41* (tonoplast intrinsic protein 41), a reference gene from *Arabidopsis*, indicated that the different lines exhibited varying expression levels for the *Nictaba*-like genes. Based on these results four transgenic lines for each *GmNLL* were selected for detailed analyses (Figure 6). It should be noted that the 35S::*NLL1* lines showed a significantly higher relative expression to *TIP41*, when compared to the 35S::*NLL2* lines.

**Figure 7:**
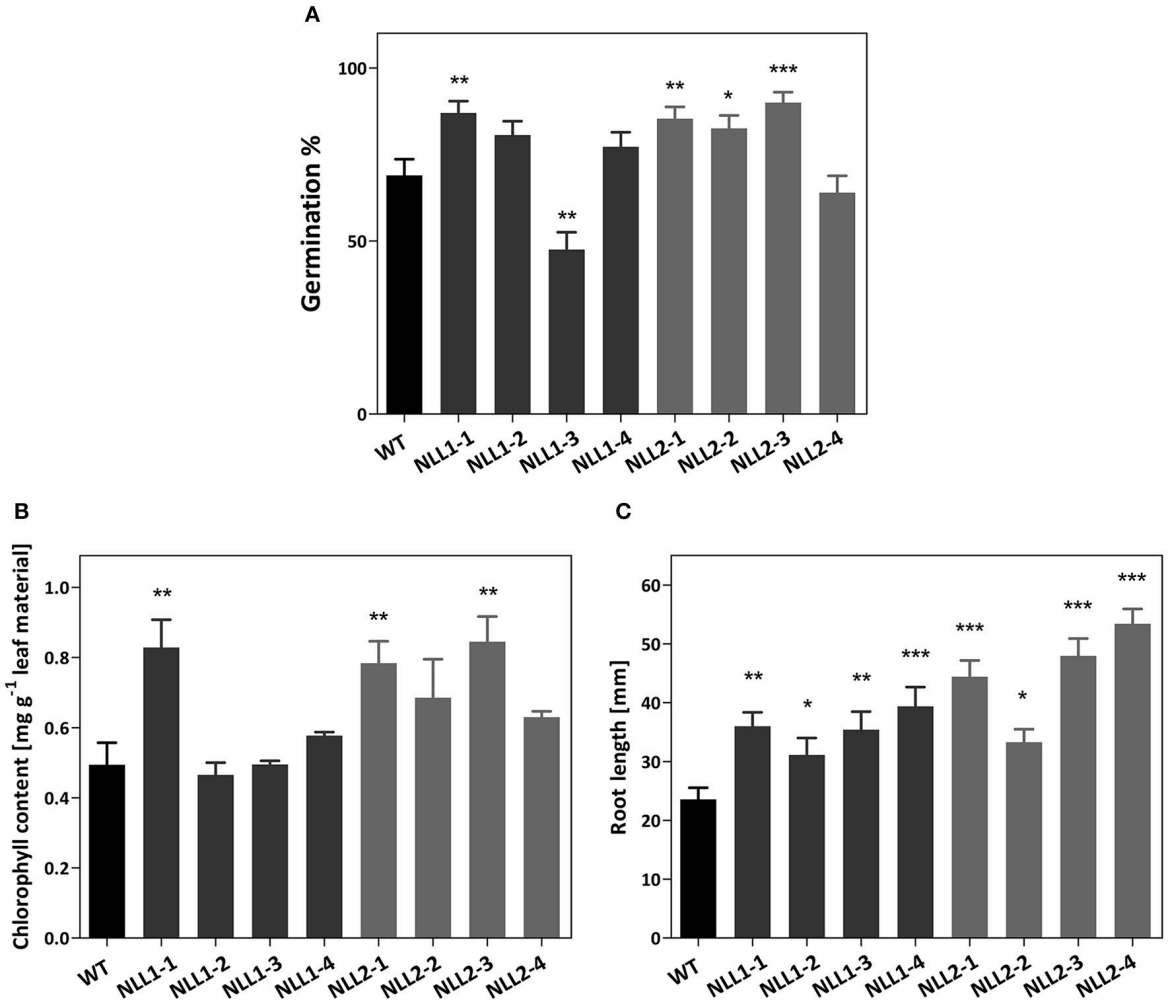
**(A)** Effect of *GmNLL1* and *GmNLL2* overexpression on *Arabidopsis* germination on ½ MS supplemented with 150 mM NaCl. Germination percentage determined on day 6, result from two biological replicates with 50 seeds per replicate, germination percentages with the corresponding standard errors are shown. **(B)** Total chlorophyll content (chlorophyll *a* + *b*) of *GmNLL1* and *GmNLL2* transgenic lines and wild type plants 7 days after transfer to ½ MS supplemented with 150 mM NaCl. Data shows the mean ± SE of two biological replicates. **(C)** Root length of 14-day-old *GmNLL1* and *GmNLL2* transgenic lines and wild type plants grown on ½ MS supplemented with 150 mM NaCl. Data shows the mean ± SE of two biological replicates with at least 30 measurements in each replicate. Asterisks indicate statistically significant differences compared to wild type (^*^p < 0.05, ^**^p < 0.01, ^***^p < 0.001).

The salt-induced expression of *GmNLL1* in soybean led us to hypothesize that *GmNLL1* might be involved in the salt stress response. In a first experiment the transgenic *Arabidopsis* lines overexpressing *GmNLL1* and *GmNLL2* were investigated for their salt stress tolerance during germination and seedling stages. Control experiments in which the germination percentage of the seeds was examined on half strength MS medium containing no salt, demonstrated that except for *NLL1-3* and *NLL2-4*, all lines exhibited the same germination percentage. Seed germination on medium containing 50 mM NaCl revealed no differences between the wild type and transgenic lines after 6 days (data not shown). On the contrary, all overexpression lines except for *NLL1-3* exhibited a similar or significantly higher germination rate on MS medium containing 150 mM NaCl compared to the wild type (Figure 7A). The lower germination percentage for *NLL1-3* and *NLL2-4* on half strength MS medium in the absence of salt could explain the lower (*NLL1-3*) or similar (*NLL2-4*) germination percentage on medium containing 150 mM NaCl.

In order to explore the effect of salt stress at the seedling stage, a second experiment was performed in which the post-germination growth was investigated. The transgenic lines overexpressing *GmNLL1* and *GmNLL2* were allowed to germinate and grow on half strength MS for 1 week, and were then transferred to half strength MS supplemented with 50 or 150 mM salt. Seven days after transfer, leaf material was harvested and chlorophyll *a* and *b* were determined to estimate leaf discoloration. Under 50 mM salt conditions, no differences in chlorophyll content could be observed between wild type and transgenic plants. However, the total chlorophyll content was significantly lower for all stress treated plants compared to those of plants that had grown on normal half-strength MS medium (data not shown). When transgenic and wild type plants were transferred to medium containing 150 mM salt, the total chlorophyll content differed significantly for some of the overexpression lines (*NLL1-1, NLL2-1, and NLL2-3)* when compared to the wild type plants (Figure 7B).

In a third experiment the effect of *GmNLL1* and *GmNLL2* expression on primary root length was examined for transgenic lines and wild type plants grown in the presence of different concentrations of NaCl (0, 50, or 150 mM). No differences in primary root length were observed between wild type plants and overexpression lines grown on the normal MS medium for 14 days, nor on MS medium supplemented with 50 mM salt. However, the primary root length of transgenic lines was significantly longer than the roots of wild type plants when plants were grown on MS supplemented with 150 mM salt (Figure 7C), suggesting that some of the *GmNLL1* and *GmNLL2* overexpression lines are more tolerant to high salt stress (150 mM NaCl) compared to wild type plants, both at the germination and the post-germination stage.

### Responsiveness of the *Arabidopsis GmNLL* overexpression lines toward aphids

**Figure 8:**
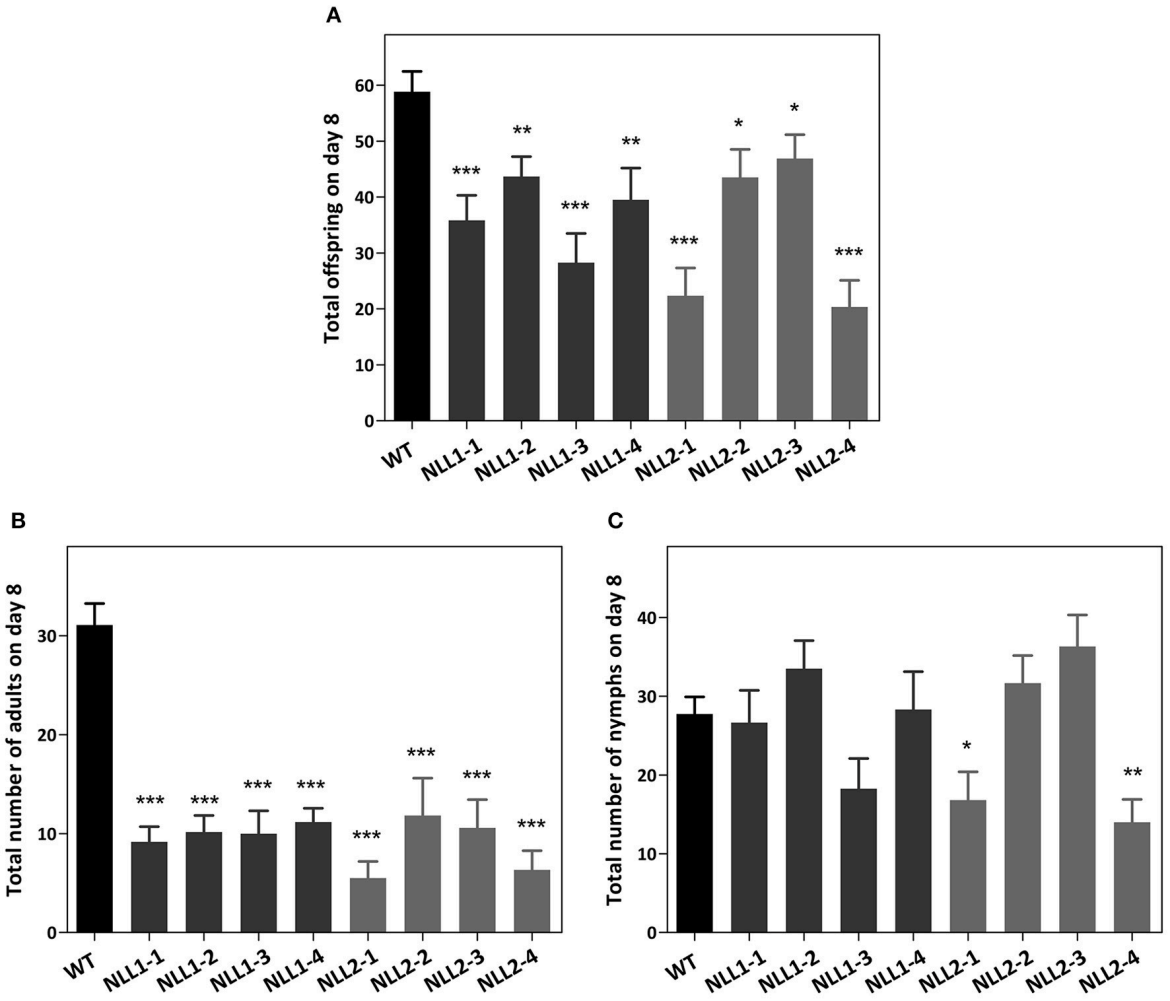
***Myzus persicae* aphid performance in a non-choice test on wild type *Arabidopsis* plants and eight transgenic lines**. The total offspring was counted after 7 days **(A)**. The number of adults **(B)** and nymphs **(C)** residing on the plants is also shown. Values are the means ± SE and represent the results from two biological replicates with six individual plants of every line in each of the replicates. Asterisks mark significant differences compared to wild type (^*^p < 0.05, ^**^p < 0.01, ^***^p < 0.001).

To confirm the role of *GmNLL* in the plant defense against aphids, transgenic lines and wild type plants were infected with *M. persicae*. The observations from the two biological experiments were reproducible and the first detrimental effect of the overexpression of *GmNLL1* and *GmNLL2* was already witnessed on day 5. All adults survived on the wild type plants, while on all overexpression lines, except for *NLL2-4*, a number of the adults had died (4.1%) or started to develop wings (7.9%), suggesting that the adults found the environment unfavorable. A clear decrease in the total number of aphids on the overexpression lines compared to the wild type plants was demonstrated after 7 days (Figure 8A). Especially fewer adults resided on all overexpression lines (Figure 8B) and for some of the overexpression lines (in particular *NLL2-1* and *NLL2-4*), there is also a significant decrease in the amount of nymphs (Figure 8C).

### Ectopic expression of *GmNLL1* and *GmNLL2* in *Arabidopsis* results in enhanced protection against *pseudomonas syringae* and does not enhance plant resistance to *Phytophthora brassicae*

Since *GmNLL1* and *GmNLL2* gene expression in soybean was significantly upregulated upon infection with *P. sojae* (Figure 5), the hypothesis was put forward that *GmNLLs* play a role in plant defense responses. The *Arabidopsis* lines overexpressing *GmNLL1* or *GmNLL2* and wild type plants were challenged with *P. brassicae* using mycelium plugs or zoospore drop inoculation to investigate the effect of *GmNLL* overexpression on the plant's resistance to pathogen infection. However, no differences in disease progression were observed between wild type plants and the *GmNLLs* overexpression lines. All plants became heavily colonized by *P. brassicae* as confirmed by staining of callose deposition in infected leaves (Results not shown).

**Figure 9:**
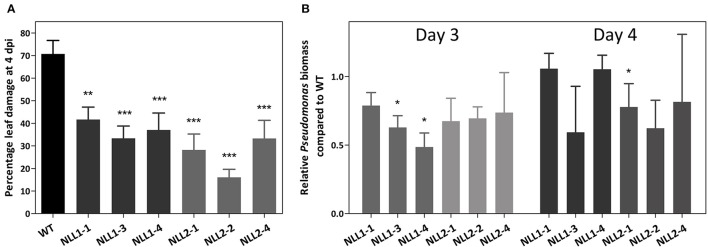
**(A)** Disease symptoms on wild type and transgenic *Arabidopsis* lines after infection with *Pseudomonas syringae*. Percentage leaf damage of infected leaves at 4 dpi was determined in Assess 2.0 and represents two biological replicates. Error bars ± SE, asterisks indicate significant differences compared to the wild type. **(B)** Relative *Pseudomonas* biomass in the overexpression lines, compared to the *Pseudomonas* biomass in wild type plants. Analysis was performed on infected leaves at 3 dpi (left panel–light gray) and 4 dpi (right panel–dark gray). qPCR data were normalized with two *Arabidopsis* reference genes in REST-384 and represents two biological replicates. Error bars ± SE, asterisks indicate significantly different ratios of the transgenic lines compared to wild type (^*^p < 0.05, ^**^p < 0.01, ^***^p < 0.001).

Wild type *Arabidopsis* plants and transgenic 35S::*GmNLL1* and 35S::*GmNLL2* plants were subjected to bacterial infection with *Pseudomonas syringae* pv. *tomato* to further investigate the role of *GmNLLs* in plant defense. Disease symptoms, bacterial growth and cell death were monitored daily. The first 2 days after the infection, no visible signs of bacterial infection were observed. Starting from 3 days post-infection, lesions were observed on the leaves and reduced disease symptoms were clear 4 days post-infection for the overexpression lines compared to the wild plants (Figure 9A and Supplementary Figure 2). In wild type plants, around 70% of the leaf is constituted of discolored lesions caused by the pathogen infection, while for all overexpression lines, the percentage of leaf damage ranged between 16 and 42% 4 days post-infection. The lesion area of mock infected plants was also measured for all time points but the calculated lesion area was never higher than 2%.

Additionally, bacterial growth of infected wild type and transgenic plants was assessed by determination of the biomass of *Pseudomonas syringae* in the inoculated *Arabidopsis* leaves. At 3 days post-infection all mean ratios for *Pseudomonas syringae* biomass in the transgenic lines are lower than those of the wild type plants (Figure 9B), but only two transgenic lines show statistically significant differences compared to the wild type plants. At 4 days post-infection, the ratios of wild type and transgenic plants were more alike and only line *NLL2-1* demonstrated a significantly lower *Pseudomonas* biomass than the wild type.

## Discussion

### A nucleocytoplasmic localization for the *Gm*NLL proteins

The two *GmNLL* genes under study are characterized by a different domain architecture. The *GmNLL1* gene encodes a Nictaba domain preceded by an N-terminal domain with unknown function while the *Gm*NLL2 sequence contains an unrelated N-terminal domain followed by two tandem arrayed Nictaba domains. Similar to the Nictaba sequence from tobacco, the NLL sequences from soybean do not possess a signal peptide, and are presumably synthesized on free ribosomes in the cytosol of the plant cell (Chen et al., 2002). Microscopic analysis of EGFP fusion proteins confirmed the presence of the *Gm*NLLs in the cytoplasm of the plant cell, but also showed fluorescence in the nucleus. The localization of the tobacco lectin in the nucleus was initially explained by the presence of a classical nuclear localization signal, required for traditional active nuclear import (Chen et al., 2002). The functionality of the nuclear localization signal was later confirmed by Lannoo et al. (2006) since transient expression of a lectin-EGFP construct with a mutation in the nuclear localization signal sequence changed the fluorescence pattern whereby the presence of Nictaba-EGFP was restricted to the cytoplasm. Recently, these results were questioned since new localization experiments with a mutated nuclear localization signal did not affect the nucleocytoplasmic localization of the fusion protein in stably transformed tobacco suspension cultures and stably and transiently transformed *N. benthamiana* leaves, indicating that the presumed nuclear localization signal is not required for translocation of Nictaba from the cytoplasm into the nucleus (Delporte, 2013). Unlike the Nictaba sequence the *Gm*NLL sequences do not contain a classical nuclear localization signal. Furthermore, *Gm*NLL-GFP fusions (approximately 47 and 75 kDa for *Gm*NLL1 and *Gm*NLL2, respectively) are too large to allow passive diffusion into the nucleus. It should be noted that additional nuclear import pathways have been characterized, depending on different import signals and these might be involved in nuclear translocation of nucleocytoplasmic lectins (Ziemienowicz et al., 2003; Pemberton and Paschal, 2005). Thus far, it remains unclear how the soybean NLL proteins are partially translocated from the cytosol to the nucleus, similar to the tobacco lectin and other nucleocytoplasmic lectins (Al Atalah et al., 2011; Van Hove et al., 2011; Delporte, 2013). Considering the confined localization of the *Gm*NLLs in the cytoplasm and nucleus, interacting partners and networks should be identified in the same cellular compartments. At present it cannot be excluded that the expression pattern would change under stress conditions, as described before for other proteins (García et al., 2010; Moore et al., 2011). Therefore, it could be interesting to investigate the localization pattern of these proteins when the plant is triggered by stress application. Expression of the GFP-NLL fusion proteins under control of their own promoter could be a convenient approach.

### *Nictaba*-like genes from soybean are stress inducible, similar to the tobacco lectin gene

The quantitative analysis of the *NLLs* in soybean at tissue level revealed a unique temporal and spatial expression pattern under normal environmental conditions. Although, there is high sequence similarity between the two *Nictaba*-like lectin sequences (29% sequence identity and 39% sequence similarity for the Nictaba domains), their unique expression profile suggests that a basal expression of the *NLL* genes in soybean is necessary for normal development of the soybean plant. These results are in contrast with the *Nictaba* gene from tobacco, which is not expressed under normal environmental conditions, suggesting that this protein has no role in normal growth or development of the tobacco plant (Chen et al., 2002). It was shown that only jasmonate treatment, insect herbivory and cold stress could trigger the expression of the *Nictaba* gene in tobacco (Chen et al., 2002; Vandenborre et al., 2009a, 2010; Delporte et al., 2011).

The results from our qPCR analysis are in accordance with the RNA-seq data reported by Severin et al. (2010). A comparative analysis for tissue-specific expression of the *NLL1-2* genes, the *SBA* gene, the *SVL* gene and the reference genes is represented in Supplementary Table 6. There are notable differences in the transcript levels of the root samples for the *NLL1* and *NLL2* gene. This discrepancy could be explained by differences between the developmental stages of the plant in both studies. Chragh et al. (2015) investigated the transcript levels of the *SVL* gene in 2-week-old plants by RT-qPCR and found significantly higher levels for *SVL* in unifoliate leaves compared to the other tissues analyzed. These observations are in line with our qPCR data of 11-day-old unifoliate leaf and root samples, and in agreement with the study of Saeed et al. (2008) in which the GUS reporter system was used to characterize the temporal and spatial expression of the *SVL* promoter in *Arabidopsis*.

Investigation of stress inducibility of the *NLL* genes demonstrated that the expression of the two *Nictaba*-like genes was induced by salt treatment (Figure 5) whereas only minor changes in *NLL* transcript levels were observed after treatment with MeJA, ABA, or SA (Supplementary Figure 1). Unexpectedly, methyl jasmonate had no effect on the expression of any of the tested *NLLs* in soybean while MeJA is one of the major triggers for the expression of *Nictaba* in tobacco (Chen et al., 2002).

Treatment with *P. sojae*, an economically important soybean pathogen, resulted in an upregulation of *GmNLL1* and *GmNLL2* (Figure 5C). These results are in agreement with the identified ESTs for *NLL1* in a cDNA isolated from *P. sojae*-infected hypocotyls (2 days post-infection; Torto-Alalibo et al., 2007). It was demonstrated that transcript levels of *GmPR10*, one of the soybean pathogenesis-related protein genes, were already upregulated 3 h post-infection (Xu et al., 2014), indicating that *NLLs* are relatively late *P. sojae*-responsive genes. Recently, several studies focused on the elucidation of the different hormone pathways that are associated with compatible and incompatible soybean-*Phytophthora sojae* interaction. At the transcriptional level, induction of the jasmonic acid pathway was shown to be involved in compatible interactions together with suppression of the ethylene pathway and no significant changes in the SA pathway were observed (Lin et al., 2014). However, recent proteomic data revealed that different components of the SA pathway were downregulated upon infection with virulent *P. sojae* (Jing et al., 2015). The specific components and their role in the complex mechanism of the soybean-*Phytophthora sojae* interaction are not completely resolved and further investigations are necessary to determine the role of the SA, ethylene and jasmonic acid pathway in this multifaceted interaction.

*Aphis glycines* infestation of soybean leaves significantly triggered the expression of *NLL1* and *NLL2*. Induction of lectin gene expression upon insect infestation was already reported for *Nictaba*. However, Nictaba accumulation in the tobacco plant was only upregulated after insect attack of the caterpillars *Spodoptera littoralis* and *Manduca sexta*, and the spider mite *Tetranychus urticae*. Infestation of aphids (*Myzus nicotianae*) or whiteflies (*Trialeurodes vaporariorum*) or infection with other pathogens (tobacco mosaic virus, *Botrytis cinerea* or *Pseudomonas syringae* pv. *tabaci*) did not alter the expression of the tobacco lectin (Lannoo et al., 2007; Vandenborre et al., 2009a,b).

Our results demonstrate that soybean *NLL* genes are responsive to both biotic and abiotic stresses. Such a crosstalk is orchestrated by the involvement of not only plant hormones, but also MAPK (mitogen-activated protein kinase), ROS (reactive oxygen species), transcription factors, heat shock factors and small RNAs and was reviewed and reported for multiple plants including soybean (Fujita et al., 2006; Atkinson and Urwin, 2012; Nakashima et al., 2014; Rejeb et al., 2014; Ramegowda and Senthil-Kumar, 2015; Gupta et al., 2016).

### Ectopic expression of *GmNLLs* in *Arabidopsis* confers plant tolerance to salt stress, aphid infestation and *Pseudomonas syringae* infection

Our data show that soybean Nictaba-like lectins confer tolerance to salt stress in *Arabidopsis* transgenic lines. To further examine the roles of *Gm*NLLs in abiotic stress tolerance, the transgenic overexpression lines and wild type plants were subjected to salt stress in multiple experimental set-ups. The data of the germination assay, post-germination assay, and root length assay indicated that overexpression of *GmNLL1* and *GmNLL2* resulted in higher tolerance to salt stress (150 mM NaCl). Nevertheless, they do not show enhanced tolerance to mild salt (50 mM) stress conditions. Noteworthy, overexpression lines *GmNLL1-1, GmNLL2-1*, and *GmNLL2-3* display the highest enhanced tolerance in all salt stress related experiments. The differences between the different lines did not correlate with the expression level of the *GmNLLs* in *Arabidopsis*. It is possible that these lines have higher amounts of *Gm*NLLs at the protein level but this could not be investigated since *Gm*NLL specific antibodies are not available. Although, the protein abundances of the *Gm*NLLs could not be determined, all overexpression lines performed better than the wild type plants in the germination and root growth experiments. The differences between the lines could be explained by a combination of post-transcriptional, translational, and degradative regulation after the expression of mRNA (Vogel and Marcotte, 2012; Feussner and Polle, 2015). Future salt stress experiments on adult *Arabidopsis* plants could be helpful to investigate whether older plants also possess these salt tolerant characteristics and if *GmNLL1* and *GmNLL2* might be components of the regulatory pathways of salt stress in plants.

Infection assays with *P. brassicae* did not show an enhanced disease resistance for the tested overexpression lines compared to wild type *Arabidopsis* plants. Bacterial blight of soybean is caused by *Pseudomonas syringae* pv. *glycinea* and can cause significant yield losses. *Arabidopsis* plants overexpressing *GmNLLs* were used in an infection assay with *Pseudomonas syringae* pv. *tomato*, an *Arabidopsis* compatible pathogen (Katagiri et al., 2002) and demonstrated that less disease symptoms were observed on the transgenic lines compared to wild type plants. These observations could be explained by reduced bacterial biomass ratios for some of the overexpression lines. It was demonstrated that *Pseudomonas syringe* induces both SA and JA pathways (Spoel et al., 2003) but RT-qPCR analysis demonstrated that these pathways are not perturbed in the *Pseudomonas* infected *GmNLL* overexpression lines (data not shown).

Overexpression of *GmNLLs* was shown to reduce aphid performance on the transgenic *Arabidopsis thaliana* lines. Since the *GmNLLs* genes are expressed constitutively, the lectin will be present in all plant tissues and will also reach the phloem. Sucking of the phloem sap is the most likely route for the lectin to enter the aphid and interact with its tissues, metabolic processes and development. The total offspring of *M. persicae* was significantly reduced in all overexpression lines, ultimately leading to a reduced population buildup. Our results clearly showed that considerably fewer adults were present on the transgenic lines. We expect that there is a combined effect of the *Gm*NLLs on survival of the aphids and in their reproduction. Future studies can focus on the mechanism(s) of the insecticidal activity. Experiments with tobacco plants indicated that *Nictaba* expression was not induced by aphid (*M. nicotianae*) feeding but insect feeding by *M. sexta, S. littoralis*, and *T. urticae* did trigger Nictaba accumulation (Lannoo et al., 2007; Vandenborre et al., 2009a,b). Furthermore, feeding experiments with transgenic tobacco plants in which the *Nictaba* gene was silenced, demonstrated that *S. littoralis* development was enhanced while overexpression of *Nictaba* led to significantly slower larval development of both *S. littoralis* and *M. sexta* (Vandenborre et al., 2010). This result confirms our hypothesis that Nictaba-like lectins from different species exhibit a strong direct insecticidal activity, but their specificity toward different insects apparently differs. Overexpression of the *GmNLLs* in *Arabidopsis* did not alter *PAD4* (phytoalexin deficient 4) transcript levels, a key component in the *Arabidopsis*-*Myzus persicae* signaling pathway (Louis and Shah, 2015; data not shown). These observations favor the role of Nictaba-like proteins in defense mechanisms rather a function in signaling pathways upon insect feeding.

All previous research from NLLs focused on the model species *Arabidopsis* and tobacco. Hence, this is the first study that focusses on NLLs in a crop species. Our data show that similar to Nictaba in tobacco, the NLLs from soybean can also be considered as stress inducible proteins. Nevertheless, the *Nictaba*-like genes in both species act differently. The expression of *Nictaba* from tobacco is increased after treatment with jasmonates whereas this is not the case for the soybean *NLLs* under study. *Nictaba* expression in tobacco was enhanced after insect herbivory by caterpillars but not by aphids. For soybean, our data clearly show that *A. glycines* infestation triggers the expression of particular *NLL* genes. Furthermore, *GmNLL* overexpression lines in *Arabidopsis* reduced the growth and development of *M. persicae*. In addition, these transgenic lines also enhanced tolerance to salt stress at the seedling stage, and showed less disease symptoms upon *Pseudomonas syringae* infection. The data strongly suggest the involvement of *Gm*NLLs in plant defense responses not only against pest or pathogens, but also in abiotic stress. These results propose that *GmNLLs* are controlled by a complex regulatory network. *Gm*NLL1 and *Gm*NLL2 are two possible candidates to further elucidate the physiological importance of the Nictaba-like lectins from soybean, which can ultimately lead to novel strategies and design of crop plants with improved tolerance to changing environmental conditions.

## Author contributions

SVH, EVD outlined and designed the study. SVH performed the experiments, analyzed and interpreted the data and prepared the manuscript. GS assisted with the design and interpretation of the aphid experiments. EVD conceived and supervised the experiments and critically revised the manuscript. All authors have read, revised and approved the final manuscript.

### Conflict of interest statement

The authors declare that the research was conducted in the absence of any commercial or financial relationships that could be construed as a potential conflict of interest.

## Abbreviations

ABA: abscisic acid
BY-2: Bright Yellow-2
EGFP: enhanced green fluorescent protein
MeJA: methyl jasmonate
Murashige and Skoog: MS
NLL: Nictaba-like lectin
SA: salicylic acid
SBA: soybean agglutinin
SVL: soybean vegetative lectin.
