# Supplementary material for: Overexpression of Nictaba-Like Lectin Genes from Glycine max Confers Tolerance toward Pseudomonas syringae Infection, Aphid Infestation and Salt Stress in Transgenic Arabidopsis Plants
Source: Front Plant Sci. 2016 Oct 25;7:1590. doi: 10.3389/fpls.2016.01590 (PMC5078610; doi:10.3389/fpls.2016.01590)
Supplement: Supplementary file 1 [file DataSheet1.DOCX]

Supplementary Material

**Overexpression of *Nictaba*-Like Lectin Genes from *Glycine max* Confers Tolerance towards *Pseudomonas syringae* Infection,
Aphid Infestation and Salt Stress in Transgenic *Arabidopsis* Plants**

**Sofie Van Holle, Guy Smagghe and Els J.M. Van Damme***

*** Correspondence:** Prof dr Els JM Van Damme: elsjm.vandamme@ugent.be

# Supplementary Figures and Tables

## Supplementary Figures


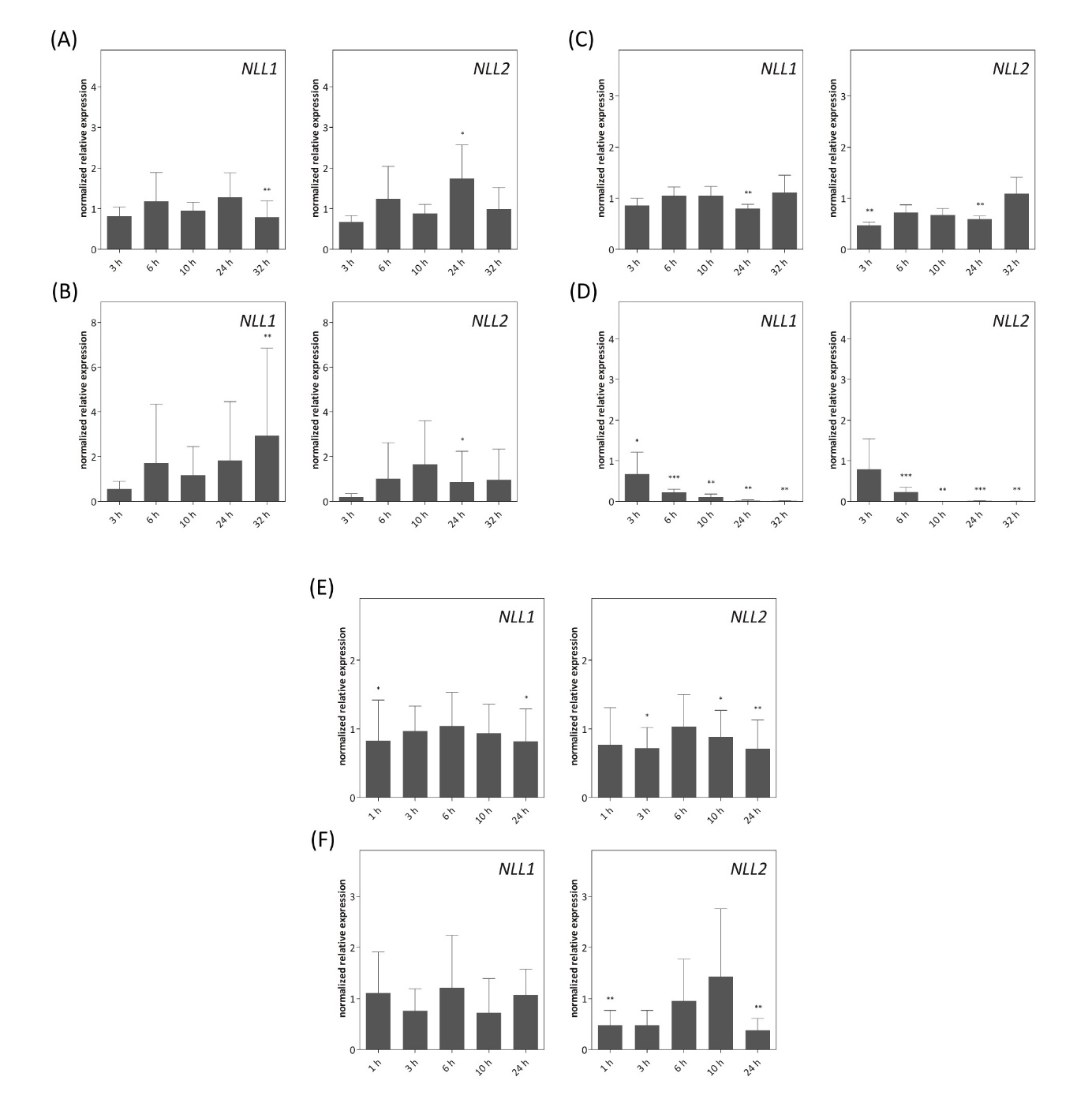


**Supplementary Figure 1.** Relative expression levels of *GmNLLs* were determined by RT-qPCR in leaf (A, C, E) and root (B, D, F) samples collected after ABA (A, B), SA (C, D) and MeJA (E, F) treatment. Three independent biological replicates have been performed. Error bars are SE and asterisks indicate significant differences compared to the control treatment at the indicated time points.


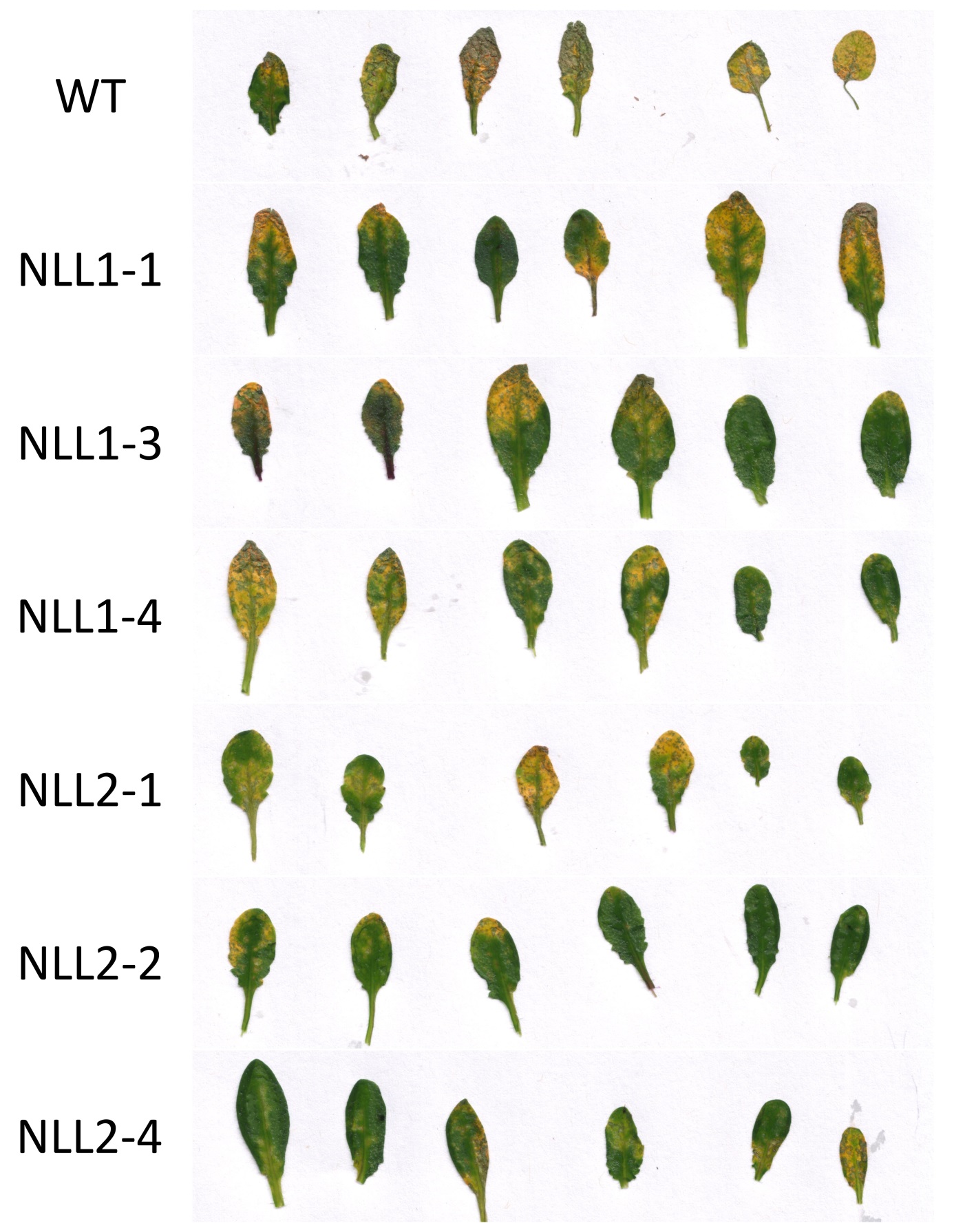


**Supplementary Figure 2.** Development of leaf chlorosis in wild type and transgenic *Arabidopsis* plants at 4 dpi after infection with *Pseudomonas syringae* pv. *tomato* DC3000.

## Supplementary Tables

Supplementary Table 1. Overview of gene specific primers

| **Target gene** | **Forward primer (5’-3’)** | **Reverse primer (5’-3’)** |
| --- | --- | --- |
| *NLL1 (Glyma.06G221100)* | CAATTTTTGCAGATTGTTGAGA | TTGGCAAATGAAGAAAACGA |
| *NLL2 (Glyma.20G020900)* | ATGGGGGCTTCACAATCAC | TTAGTTCTTTGGTTTGATGACAATG |

Supplementary Table 2. Overview of primers used in molecular cloning

| **Target gene/sequence** | **Primer** | **5’-3’ sequence** |
| --- | --- | --- |
| attB1 and attB2 adaptor sites | evd 2 | GGGGACAAGTTTGTACAAAAAAGCAGGCT |
|  | evd 4 | GGGGACCACTTTGTACAAGAAAGCTGGGT |
| Forward gene specific *NLL1* primer containing part of the attB1 site | evd 1022 | AAAAAGCAGGCTTCACCATGCCTTTCAAGAAGCCTCA |
| Reverse gene specific *NLL1* primer without stop codon and containing part of the attB2 site | evd 1023 | AGAAAGCTGGGTGAGTTAAAGGTTTGATGAGGG |
| Forward gene specific *NLL2* primer containing part of the attB1 site | evd 1024 | AAAAAGCAGGCTTCACCATGGGGGCTTCACAATCAC |
| Reverse gene specific *NLL2* primer without stop codon and containing part of the attB2 site | evd 1025 | AGAAAGCTGGGTGGTTCTTTGGTTTGATGACAATG |
| Reverse gene specific *NLL1* primer containing part of the attB2 site | evd 1032 | AGAAAGCTGGGTGTTAAGTTAAAGGTTTGATGAGGG |
| Reverse gene specific *NLL2* primer containing part of the attB2 site | evd 1033 | AGAAAGCTGGGTGTTAGTTCTTTGGTTTGATGACAATG |

Supplementary Table 3. Overview of gene specific primers used for RT-qPCR

| **Target gene** | **Forward primer (5’-3’)** | **Reverse primer (5’-3’)** |
| --- | --- | --- |
| *NLL1 (Glyma.06G221100)* | AACCCTGGTGAAACCTTGAA | TTTCCGCTCCACACTTCATA |
| *NLL2 (Glyma.20G020900)* | TGCCAACAACACCAATTCTT | TCGTGCCACTTGCTTCTTTA |
| *SBA (Glyma.02G012600)* | CTTGGGATCCACCAAATCC | GTTGGCCAAATCCCAAGAC |
| *SVL (Glyma.02G156800)* | CGTTGAAACCCATGATGTGA | TGAGCACAAAGCTTGGAAGA |
| *UKN1 (Glyma.12G020500)* | TGGTGCTGCCGCTATTTACTG | GGTGGAAGGAACTGCTAACAATC |
| *SKIP16 (Glyma.12G051100)* | GAGCCCAAGACATTGCGAGAG | CGGAAGCGGAAGAACTGAACC |
| *Act11 (Glyma.18G290800)* | ATCTTGACTGAGCGTGGTTATTCC | GCTGGTCCTGGCTGTCTCC |
| *60s (Glyma.13G318800)* | AAAGTGGACCAAGGCATATCGTCG | TCAGGACATTCTCCGCAAGATTCC |
| *ABC (Glyma.12G020500)* | GATCAGCAATTATGCACAACG | CCGCCACCATTCAGATTATGT |
| *Fbox (Glyma.12G051100)* | AGATAGGGAAATTGTGCAGGT | ctaatggcaattgcagctctc |
| *IDE (Glyma.03G137100)* | atgaatgacggttcccatgta | ggcattaaggcagctcactct |
| *Act2 (AT3G18780)* | GATGAGGCAGGTCCAGGAATC | GTTTGTCACACACAAGTGCATC |
| *PEX4 (AT5G25760)* | TGCAACCTCCTCAAGTTCG | CACAGACTGAAGCGTCCAAG |
| *OprF (PSPTO_2299)* | AACTGAAAAACACCTTGGGC | CCTGGGTTGTTGAAGTGGTA |

Supplementary Table 4. Reference genes used in the stress experiments

| **Experiment** | **Reference genes** | **Reference** |
| --- | --- | --- |
| Growth experiment | *Act11, SKIP16, UNK1* | Hu et al., 2009 |
| *Aphis glycines* infestation | *Act11, SKIP16, IDE* | Libault et al., 2008; Hu et al., 2009 |
| *Phytophthora sojae* infection | *Act11, SKIP16, IDE* | Libault et al., 2008; Hu et al., 2009 |
| NaCl treatment | *60s, SKIP16, Fbox* | Hu et al., 2009; Le et al., 2012 |
| ABA treatment | *60s, ABC, Fbox* | Le et al., 2012 |
| JA treatment | *Act11, SKIP16, UNK1* | Hu et al., 2009 |
| SA treatment | *Act11, SKIP16, UKN1* | Hu et al., 2009 |

Supplementary Table 5. Mean Cq value of the reference genes and the *GmNLL* genes in all analyzed tissues

| **Gene** | **Mean Cq ± Stdev** |
| --- | --- |
| *Act11* | 20.1 ± 2.2 |
| *UKN1* | 22.4 ± 2.9 |
| *SKIP16* | 20.7 ± 2.4 |
| *NLL1* | 21.1 ± 3.8 |
| *NLL2* | 26.0 ± 3.7 |

**Supplementary Table 6.** RPKM normalized tissue specific expression of *NLL1*, *NLL2*, *SBA*, *SVL* and reference genes downloaded from Soyseq (http://soybase.org/soyseq/) RPKM: reads/kilobase/million; DAF: days after flowering (Severin et al., 2010)

| **Gene** | **young leaf** | **root** | **one cm pod** | **pod shell 14 DAF** | **seed  14 DAF** | **seed  28 DAF** | **seed  35 DAF** | **seed  42 DAF** |
| --- | --- | --- | --- | --- | --- | --- | --- | --- |
| *NLL1* | 14 | 51 | 19 | 51 | 2 | 2 | 3 | 2 |
| *NLL2* | 5 | 15 | 8 | 6 | 3 | 2 | 1 | 2 |
| *SBA* | 0 | 0 | 0 | 0 | 7 | 2235 | 6638 | 7071 |
| *SVL* | 3003 | 1 | 747 | 2323 | 2 | 5 | 8 | 2 |
| *UKN1* | 13 | 15 | 16 | 25 | 16 | 6 | 9 | 5 |
| *SKIP16* | 23 | 49 | 25 | 27 | 24 | 7 | 13 | 9 |
| *Act11* | 34 | 37 | 35 | 23 | 20 | 13 | 26 | 13 |
